# Current News and Opinion

**Published:** 1888-02

**Authors:** 


### Current News and Opinion.

### DENTAL SQUIBS.

BY "MEANDER."

In a recent number of one of our dental journals there were five "original communications" on "Capping Exposed Pulps," "Treatment of Exposed Pulps," and one brother got so far as to adopt quills He has perception enough to see that it won't do to put any kind of permanent pressure on an exposed, or even on a partially exposed, pulp.

Every one who practices dentistry would like to succeed. As one element of success, how would kindness work ? Kindness so marked as to germinate into sympathy. An honest, cordial reception will establish a more enduring relation, and that is what is wanted to retain patients.

Gentleness, politeness, unaffected cordiality, cleanliness, and courtesy are five nouns that will grace any dental office in this or any other land.

Some of our wise brethren advocate the combination of gold foil with tin foil, rolled together, as a good filling material for—let Meander add *soft*—teeth. It is a most excellent marriage, receiving the blessing of all conservative relatives. It forms an excellent commencement for cervico-approximating walls. Bury the tin, though ! That is, in such cases, put over the baser metal, two sheets of No. 4 gold foil.

One good brother says he uses No. 4 tin with No. 4 gold. Meander has conceived and brought forth this fact; that No. 2 tin foil will much better permit the hiding of the gold than will No. 4, and at the same time fully perform all the functions of a soft-hard filling.

But where can one get No. 2 tin foil ? Meander could find none at the depots, therefore he wrote to Edward Kearsing, of No. 101 Hoyt St , Brooklyn, N. Y., and this Beater beat him out some good, pure, reliable No 2 tin foil. This same Beater beats out all of Meander's gold foil, and has done so these many years !

---

### ANTISEPTIC MOUTH WASHES.

*Editor Independent Practitioner :*

Being convinced that not only decay of the teeth, but many of the complications following upon it, as well as many affections of the mucous membrane of the mouth, of the gums and of the pericementum, are of parasitic origin, you may readily understand that I should attach great value to any substance possessing marked antiseptic action which might be used freely in the human mouth without danger of injury to the teeth, the mucous membrane or the general health. I hope soon to be able to give you the results of a series of experiments which I have been making with reference to this question; at present I wish only to call attention to a formula which I published some two years ago in a German medical journal, and which I see has found its way into various dental journals. This formula is

| | |
|---|---|
| Thymol, | 0.25 |
| Benzoic acid, | 3.00 |
| Tincture of Eucalyptus, | 15.00 |
| Water, F. | 50.00 |

This mixture possesses powerful antiseptic properties, but was never designed for use in the mouth. It was only to serve as a base upon which a good mouth wash might be constructed. I will send you, in two or three weeks, a detailed account of the experiments which I have made upon this question, and the results at which I have arrived, and hope you may be able to give them a place in the INDEPENDENT PRACTITIONER. W. D. MILLER.

---

### CORRECTION.

*Editor Independent Practitioner:*

### ROYAL COLLEGE OF DENTAL SURGEONS, OF ONTARIO.

The third annual dinner of the faculty and students of the Toronto School of Dentistry was held at the Rossin House, Toronto, Tuesday evening, Dec. 13, 1887. The class, which numbers forty-eight, is an unusually intelligent one, and the dinner was a marked success. Perhaps the most significant speech of the evening was that of the Vice-Chancellor of Toronto University, who announced that for some years the school of dentistry had been carefully watched by the Governors of the University, and so thoroughly were they convinced of the high character of the instruction afforded, and the unequivocal claims to recognition of the school, that it had been determined to accept its offer and to incorporate it as a department of Toronto University.

---

### MARRIED.

Tuesday, December 27, 1887, at Chicago, DR. LOUIS OTTOFY and MISS NELLIE L. FREEMAN.

The INDEPENDENT PRACTITIONER tenders its compliments to the happy couple, and hopes that both may live to celebrate many, many happy anniversaries.

### MISSISSIPPI VALLEY DENTAL ASSOCIATION.

CINCINNATI, January 18, 1888.

The forty-fourth annual meeting of the Mississippi Valley Association of Dental Surgeons will be held in Cincinnati on the first Wednesday in March, 1888, (March 7th).

The following is a partial list of papers to be read and subjects for discussion :

"The Dental Pulp." Junius E. Craven, D. D. S.

"Immediate Root Filling." H. A. Smith, D. D. S.

"Implantation," with clinical demonstration. M. H. Fletcher, D. D. S., M. D.

"Combination Fillings." H. J. McKellops, D. D. S.

"Constitutional Aspects of Pyorrhœa Alveolaris." J. H. Callahan, D. D. S.

"Incidents of Office Practice."

"Voluntary Papers," are expected from Dr. W. D. Miller, Berlin, Germany, and Dr. N. W. Williams, Geneva, Switzerland.

Prize essay subject. "The Causes of Deposits on the Teeth and Methods of Removing the Same." (A prize of twenty-five dollars was offered for the best essay on this subject at the last meeting of the society. See minutes for terms of awarding the same.)

Presid't A. W. HARLAN, Chicago

Sec A. G. ROSE,

Cincinnati.

C. M. WRIGHT,

Ch'm'n Ex. Committee.

---

### ST. LOUIS DENTAL SOCIETY.

The St. Louis Dental Society held its annual meeting Tuesday evening, Jan. 3d, and elected the following officers for 1888 :

*President*—Dr. Henry Fisher.

*Vice-President*—Dr. J. Warren Wick.

*Corresponding Secretary*—Dr. Wm. Conrad.

*Recording Secretary*—Dr. J. H. Spalding.

*Treasurer*—Dr. A. J. Prosser.

*Publication Committee*—Drs. A. H. Tuller, Geo. P. Holmes, W. N. Morrison.

*Committee on Elections and Ethics*—Drs. J. B. Newby, W. H. Eames, G. A. Bowman.

WM. CONRAD, Cor. Sec.
Hotel Beers.

---

### MASSACHUSETTS DENTAL SOCIETY.

The following members were elected officers for the ensuing year at the annual meeting held Dec. 9, 1887 :

*President*—H. C. Merriam, Salem, Mass.

*First Vice President*—G. A. Gerry, Lowell, Mass.

*Second Vice-President*—R. R. Andrews, Cambridge, Mass.

*Secretary*—G. F. Eames, 62 Trinity Terrace, Boston.

*Treasurer*—Edward Page, Boston.

*Executive Committee*—E. B. Hitchcock, A. H. Gilson, W. E. Page, J. K. Knight, E. C. Leach.

G. F. EAMES, Secretary.

THE FIRST DISTRICT DENTAL SOCIETY MEETING

The editor of this Journal was unable, because of illness, to attend the New York meeting of the past month, but learns that it was a success. The elaborate programme was well followed, and the clinics at the New York College of Dentistry were very instructive and well attended. Some of the papers read at the evening meetings were of decided interest, and the discussions were animated and earnest. Wednesday, January 18th, at 4 P. M., the annual dinner was given at Morrello's, about seventy persons being present. The occasion was enlivened by toasts and speeches, and was a very enjoyable affair.

---

THE SOUTHERN DENTAL JOURNAL publishes the particulars of a truly remarkable case of implantation, as follows :

Case 1. Rev. George Washington Thompson (colored). Age 52. Implanted September 26th, a right superior central. There was no *visible* inflammation at any time. At the expiration of two weeks the ligatures were removed, and in one month the tooth was formed in an old box which had not been opened for seventeen years It had turned a few shades darker.

We rise for information and a few additional particulars. Was the Rev. Geo. W. Thompson the implanter or the implantee ? If the latter, was the tooth implanted in his box or in his mouth; or are the two terms, as here used, synonymous ? If the latter be the case, are we to understand that the colored man, and he a Reverend too, had not opened his mouth for seventeen years ? And about that "*visible* inflammation." Do Reverends, or colored people, or reverend colored people, have visible inflammations ? We have seen visible indications, but not the inflammation itself, for that is supposed to be but a pathological condition. Altogether, we fear we are worse mixed over this case than was the author of "Translated from the German" in the same number, when attempting to give information concerning "Zahnhrenstlers."

---

ALBERT P. BRUBAKER, M. D., December 19, 1887, read before the Philadelphia Neurological Society a paper upon "Dental Irritation as a Factor in the Causation of Epilepsy," in which he says:

"The object of this paper is to direct the attention of physicians to a cause of epilepsy which has not hitherto been estimated at its full value, inasmuch as in none of the standard works upon neurology is the subject ever alluded to, viz., pathological states of the dental structures. That dental inflammation and disorders are more often provocative of epileptic seizures than is commonly supposed, appears quite certain from the following cases, and also from the character of the cause and its effect. Many reasons might be given why dental disorders are peculiarly adapted to call forth this periodical discharge, and why these disorders are habitually overlooked by the physician, but they need not be detailed here"

Then follows the details of sixteen cases in which dental disorders were found associated with epilepsy, the removal of the offending teeth or the placing of the oral cavity in a healthy condition being followed by a cessation of the epileptiform attacks. The paper is published in the *Medical and Surgical Reporter* of January 21st.

The Kansas State Dental Association has given some of the most important and comprehensive mechanical clinics for its members of any society of which we have knowledge. Two years ago Dr H. W. Howe, an accomplished mechanician, demonstrated before it the making of a full banded gold plate, from the refining of the gold to the final finishing. This year Dr. H. W. Parsons, of Wamego, the supervisor of clinics, issues a circular inviting all members to bring casts of any abnormal conditions, with models of the appliances used, and also to present malformations and surgical cases for comparison and instruction, and thus to found an annual museum. Such a thing might be made very interesting and profitable.

---

We learn from the Independent Practitioner that sulphuric acid contains no sulphur. We thought the formula was $H_2SO_4$. Oil of turpentine was formerly thought to be Oleum Terebinthinæ, and kerosene *has* been spoken of as Petroleum. We live to learn. *Dental Review.*

We hope you do not live in vain. A true oil is a salt. It consists of a fatty acid uniting with a glycerine base. Petroleum (*Petra-Oleum*, Rock Oil), is a misnomer. It is a hydro-carbon. It is unsafe to judge of the character of a compound by its popular name. Sulphuric acid is called oil of vitriol; but it is several degrees removed from the fats. The paragraph to which the *Review* refers was a clipping from another journal to which we intended to give credit, but the editor proposes and the compositor disposes.

---

Pliny the younger relates that Marcus Curius, nicknamed *Dentatus*, had all his teeth at birth. Richard III, had the same, and Jacobi reported the case of a Spanish dwarf, who was born with all his teeth, all of which remained; he had a beard at seven years of age, and became a father at ten. A woman named Mary Wood, aged ninety-eight, had nine new molars at that age, and a certain Scotch farmer lost all his teeth at sixty, and six months later he cut a new set without the aid of the dentist, and had them all when he died at ninety-six years of age.—*Medical Press.*

Some of the assertions in this paragraph should, we think, be taken *cum grano salis.*—Editor.

---

Margaret Dunn brought suit recently against Dr. F. Hasbrouck, of New York, to recover the snug sum of $5,000 damages for injury done to her jaw. She said that one of Dr. Hasbrouck's assistants extracted several teeth for her over two years ago, and since that time she had been unable to work her jaw, without discomfort. Experts were present and testimony given for both plaintiff and defendant. The jury decided in favor of defendant, so the woman got no pin money. A sensible set of men were the jurors.

---

Dr. Grant Bey, in the *New York Medical Record*, for November 26th, asserts that we have a more or less continuous history of medicine for nearly six thousand years, which would place the beginning of medicine at about 4,000 B. C., and as this is about the date that bible commentators have agreed in assigning to the creation of the earth, it at once shows that medicine has a very respectable age.—*Maryland Medical Journal.*

A YOUNG MOTHER with her three-and-a-half years old daughter occupied a seat in a railway car, and directly behind them sat two gentlemen engaged in conversation. In the course of their remarks one of the gentlemen said that he had not been feeling well of late, and described his ailings to his companion. At this point, our little "tot," who, unnoticed, had been quietly looking out of the window, climbed upon the seat, turned about and facing the complainant, said: "Wot oo' want is a dood dose of 'oobarb."

"I believe you are right," responded the gentleman, "and I think I will follow your advice," when both indulged in a hearty laugh at this unlooked for and sensible suggestion. F.

---

DR. B. C. WINDLE, in a paper read before the British Dental Association on "Man's Lost Incisors," reaches the following conclusions :

1. Man's original dentition included six incisors.
2. The lost incisor is the second lateral.
3. This loss is consequent upon the contraction of the anterior part of the jaw.
4. Suppression of the present lateral incisors is now taking place.
5. Conical teeth are a reversion to the primitive type.

---

THE EDITOR OF THE DENTAL ADVERTISER, of Buffalo, has a very singular way of exhibiting his appreciation of professional courtesies shown him He should have learned long ere this that men imbued with a professional spirit do not carry on a scientific discusion or attempt to advance professional knowledge by the use of personalities and scurrility. The *tu quoque* argument is seldom appealed to when any other is at hand.

---

DR. WARDWELL'S rubber foot pad for the treadle of the dental engine is something more than a mere convenience. We have one in use, and find that the engine really is made to run more easily, for the foot keeps any position on the treadle, and there is no energy wasted by slipping and sliding. It looks neat, and the engine is readily run from any position.

---

DR. A. W. HARLAN, editor of the *Dental Review*, with his wife, sailed for Europe January 26th, for a vacation trip We hope it may be marred by no untoward event, and that the net results may be health, pleasure and a renewed energy for the duties which will await his return.

---

VICK'S FLORAL GUIDE is a thing of beauty. That man has won a proud position who can claim to be first in his chosen field, and the pre-eminence of Vick in floriculture is almost universally acknowledged. Send to him at Rochester, N. Y., for flower or garden seeds, or for plants and bulbs, if you wish those that are true to name and sure to germinate.

---

DRS. BOGUE AND DAVENPORT, of Paris, have removed from No. 39 to No. 73 Boulevard Haussmann, as may be learned by a reference to their card in the advertising pages.

PARKE, DAVIS & CO. send us a fine picture of Sir Morell Mackenzie, the specialist who has won fame through his connection with the case of the Crown Prince of Germany. He seems to have been more fortunate than some of America's physicians who attained only notoriety through their connection with the cases of Presidents Garfield and Grant.

---

ARCHIVES OF DENTISTRY, in its December number, announces that it will no longer be published by J. H. Chambers & Co , the book and journal publishers of St. Louis. For the future it will be in the hands of the dentists who have so successfully edited and conducted it during the past year. We trust it may meet with continued prosperity.

---

ANOTHER of Professor N. S. Shaler's notable articles on the Surface of the Earth appears in *Scribner's Magazine* for February under the title of "Volcanoes." Among the illustrations are a number of very picturesque views of the great eruption in the Sandwich Islands, which have never before been engraved.

---

SUBSCRIBERS WHO MAKE REMITTANCES will find their receipts enclosed in the next number of the Journal. It is not convenient for us to send them by separate mail, as the account is kept with the subscription list Remember that all subscriptions should be sent to the Buffalo office.

---

DR WILLIAM STRANGER, in the *British Medical Journal* for November 25th, reports a case of gangrenous abscess of the lung caused by the stump of a tooth, which passed into the right bronchus during an extraction of several roots under chloroform, by a dentist.

---

THE MEDICAL SOCIETY OF THE STATE OF NEW YORK will hold its eighty-second annual meeting at Albany, on the 7th, 8th, and 9th of February, inst. The published programme shows a long list of papers by some of the most eminent of the physicians of the State

---

MR. PENFOLD exhibited before the Odontological society of Great Britain a new antiseptic, called by its discoverer, Salufer. It is a fluosilicate of sodium, and it is claimed that it is non-poisonous, non-irritant, but powerfully disinfectant.

---

DR. JULIAN W. RUSSELL, of Brooklyn, N. Y , is authorized to receive advertisements for the INDEPENDENT PRACTITIONER, and any contracts which he may make will be acknowledged by the publishers.

---

DR. W. D. MILLER, of Berlin, has removed from No. 2 Hausvoigteiplatz to No. 32 Voss Strasse, where those who desire to communicate with him should address their letters.

---

LUBRICANT FOR BRASS.—One part of pure india rubber melted, and two parts of vaseline, is said to make an excellent lubricant for brass. It is non-corrosive and lasting.

